# The Bacterial Compositions of Nasal Septal Abscess in Patients with or without Diabetes

**DOI:** 10.3390/life12122093

**Published:** 2022-12-13

**Authors:** Chih-Wei Luan, Ming-Shao Tsai, Yao-Te Tsai, Cheng-Ming Hsu, Chia-Yen Liu, Yao-Hsu Yang, Ching-Yuan Wu, Geng-He Chang

**Affiliations:** 1 Department of Otorhinolaryngology-Head and Neck Surgery, Lo Sheng Sanatorium and Hospital Ministry of Health and Welfare, New Taipei City 242, Taiwan; 2 Department of Otolaryngology, Chang Gung Memorial Hospital, Chiayi 613, Taiwan; 3 General Education Center, Lunghwa University of Science and Technology, Taoyuan City 333, Taiwan; 4 Graduate Institute of Clinical Medical Sciences, College of Medicine, Chang Gung University, Taoyuan City 333, Taiwan; 5 Health Information and Epidemiology Laboratory of Chang Gung Memorial Hospital, Chiayi 613, Taiwan; 6 Faculty of Medicine, College of Medicine, Chang Gung University, Taoyuan City 333, Taiwan; 7 School of Traditional Chinese Medicine, College of Medicine, Chang Gung University, Taoyuan City 333, Taiwan; 8 Department of Traditional Chinese Medicine, Chang Gung Memorial Hospital, Chiayi 613, Taiwan; 9 Head and Neck Infection Treatment Center, Chang Gung Memorial Hospital, Chiayi 613, Taiwan

**Keywords:** nasal septal abscess, hyperglycemia, diabetics, pathogen, *Klebsiella*

## Abstract

The nasal septal abscess (NSA) is a rare but potentially fatal disease causing intracranial infection. Treatments for NSA include antibiotics, surgical incision and drainage. Diabetes mellitus (DM) is a risk factor for NSA. Therefore, we assessed the pathogenic bacterial composition of NSA in diabetic patients. We analyzed the Chang Gung Memorial Hospital database to collect 79 NSA patients who received surgical incisions and drainage from 2004 to 2015. We divided them into DM and non-DM groups for analysis. We integrated the bacteria cultured from each patient, listed the top three with the highest frequency and divided the bacterial species into facultative anaerobes or aerobes and anaerobes. The microbiological cultures revealed mono-microbial infection in most of the cases. The top three facultative anaerobes or aerobes with the highest frequency of NSA-DM were *Klebsiella pneumoniae* (37.5%), methicillin-sensitive *Staphylococcus aureus* (MSSA; 25%) and methicillin-resistant *Staphylococcus aureus* (MRSA; 12.5%). The top three for NSA-non-DMs were MSSA (24%), MRSA (20%) and *Pseudomonas aeruginosa* (16%). The top three anaerobes causing NSA were *Prevotella intermedia* (25%), *Peptostreptococcus* species (12.5%) and *Propionibacterium acnes* (12.5%) in DM patients. The top three in non-DM patients were *P. intermedia* (25%), *P. acnes* (16.7%) and *Fusobacterium nucleatum* (12.5%). When treating NSA in diabetic patients, clinicians should choose empirical antibiotics for *K. pneumoniae* and *P. intermedia*, and when treating patients with NSA-non-DM, MSSA and *P. intermedia* should be considered first.

## 1. Introduction

Nasal septal abscess (NSA) is a rare bacterial infection in the space between the septal cartilage and perichondrium [1], which is usually seen in patients with nasal trauma complicated with septal hematoma [2]. The clinical symptoms of NSA showed progressive nasal obstruction and nasal pain, and the physical examination could find swollen septum mucosa [3]. Our previous research reported that diabetes mellitus (DM) is an independent risk factor for NSA in patients undergoing nasal septoplasty [1]. Moreover, the higher the severity of DM (expressed as Diabetes Complications Severity Index), the higher risk of the NSA [1].

NSA could progress to cause intracranial infection [4]. Therefore, early detection with timely aspiration or incision and drainage and the accurate selection of empirical antibiotics are crucial for preventing consequent lethal complications. To date, studies discussing the pathogens of NSA are insufficient and contain a small sample size [2,5,6]. Moreover, no study was documented on the bacterial compositions of NSA in diabetic patients. Therefore, this study included NSA patients from Chang Gung Memorial Hospital in Taiwan to compare the bacterial composition of NSA patients with diabetes and those without diabetes.

## 2. Materials and Methods

### 2.1. Study Population—NSA in Patients with or without DM

The Chang Gung Hospital group is the most extensive medical system in Taiwan, covering the main administrative regions of Taiwan from north to south, including Taipei, Linkou, Taoyuan, Chiayi, Kaohsiung, Keelung and Yunlin CGMHs. The Chang Gung research database (CGRD) contains the medical data of all hospitals in the Chang Gung system and is provided to physicians for research. Several previous studies have mentioned that the results of the retrospective analysis from Chang Gung multi-institutional hospitals were similar to that of Taiwan’s national condition [7,8,9].

We used CGRD to select the patients with NSA who were received for incision and drainage of abscess between 1 January 2004 and 31 December 2015. All the bacterial cultures were performed at the same time that NSA was diagnosed and almost at the same time that empirical antibiotics were administered. To avoid contamination of normal flora in the nose, a standard nasal clean with betadine and normal saline was performed before surgical drainage and aspiration. The pus culture can be cultured in two ways. One is directly from the surgical procedure, which first cuts the septum mucosa and then cultures the bacterium by collecting pus flowing out from the cutting edge with culture sticks; the other is needle aspiration, in which the abscess was aspirated from the nasal septum using a number 18 needle. Exclusion criteria were having an ICD-9-CM diagnostic code associated with malignancy of the paranasal sinuses or nasopharynx (ICD-9-CM: 147 and 160). We divided the patients into NSA-DM group and NSA-non-DM group according to their diabetes-related diagnostic codes (ICD-9-CM: 250, diagnosed at one inpatient or three outpatient visits). We compared the differences in the bacterial composition of NSA between the two groups.

The Institutional Review Board (IRB) of the CGMH approved this study (IRB number: No. 201900520B0).

### 2.2. Medical Comorbidities

We analyzed the major medical diseases, including hypertension, coronary arterial disease (CAD), cerebral vascular accident and chronic obstructive pulmonary disease (COPD) [10,11,12], to assess the medical comorbidities of these two groups and explore the confounding factors. The criteria for judging the above diseases were based on the medical records of these patients, the diagnosis of outpatient treatment more than three times, or the diagnosis of inpatient treatment more than once.

### 2.3. Classification and Analysis of Pathogenic Bacteria

According to the previous studies [7,9,13], we classified the cultured bacteria into two groups: facultative anaerobes, such as *Staphylococcus aureus*, or aerobes, such as *Pseudomonas aeruginosa* and anaerobes, such as *Prevotella intermedia* for analysis. Usually, antibiotics are selected based on facultative anaerobes and aerobes in cultures, although anaerobes need to be treated together [2,14]. We analyzed the top three common pathogenic bacteria that covered genus and species among the patients with NSA-DM and NSA-non-DM [7,9,13].

Moreover, we analyzed the number of bacteria isolates cultured in each NSA patient, such as mono-microbial, dual-microbial and poly-microbial infections. The results were expressed as (1) total bacteria and (2) facultative anaerobes or aerobes and anaerobes [13].

### 2.4. Assessment of Prognosis

We analyzed the intracranial infection in these two groups of NSA patients and the number of deaths despite treatment. We also examined the patients who required surgical repair for a septal perforation after NSA.

### 2.5. Statistical Analysis

Differences in gender and age between NSA-DM and NSA-non-DM patients were analyzed by Pearson’s chi-squared test, and medical comorbidities were analyzed by Fisher’s exact test. In all statistical analyses, *p*-values less than 0.05 were considered significant differences. All the analyses were performed using SAS software version 9.4 (SAS Institute, Cary, NC, USA).

## 3. Results

### 3.1. Cases Included in the Analysis

**Figure 1:**
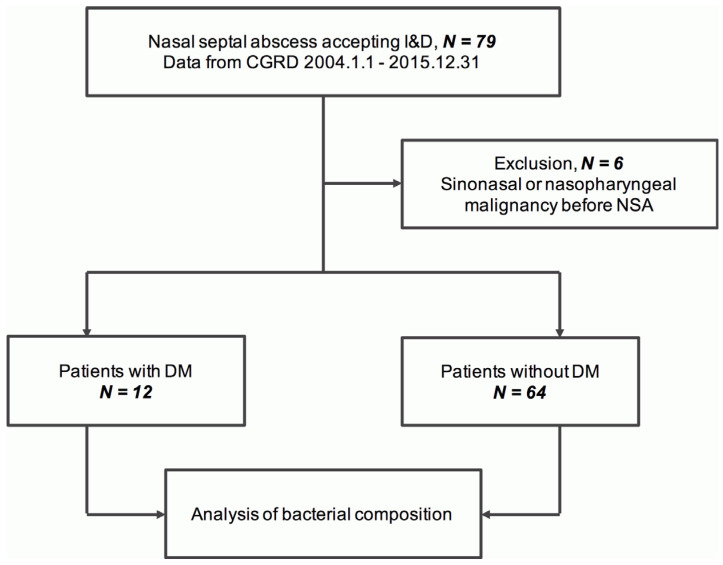
Enrolment and classification of NSA cases. Abbreviations: CGRD, Chang Gung Research Database; NSA, nasal septal abscess; DM, diabetes mellitus.

A total of 79 patients who presented with NSA were identified from the CGRD. Among them, 3 cases with histories of sinonasal or nasopharyngeal malignancy were excluded. Then, 12 patients were classified as NSA-DM and 64 as NSA-non-DM group (Figure 1).

### 3.2. Demographic Characteristics and Medical Comorbidities

The demographic characteristics and medical comorbidities among the two groups are shown in Table 1. Although no statistically significant difference was observed in gender between the two groups, the proportion of males was much higher than that of females (NSA-DM, male vs. female: 83.3% vs. 16.7%). Similarly, no significant difference in age was observed between the two groups of patients. However, the proportion of older patients was higher in the NSA-DM group (≥50 vs. <50 years: 66.7% vs. 33.3%), while in the NSA-non-DM group, the younger patients were in the majority (≥50 vs. <50 years: 35.9% vs. 64.1%). Regarding the medical comorbidities, a significantly higher proportion of hypertension and CVA was identified in the NSA-DM group (hypertension in NSA-DM vs. NSA-non-DM: 50% vs. 6.3%, *p* = 0.001; CVA in NSA-DM vs. NSA-non-DM: 33.3% vs. 1.6%, *p* = 0.002). However, the other comorbidities, including CAD and COPD, were not statistically significant differences.

### 3.3. Bacterial Cultures and Growths

**Figure 2:**
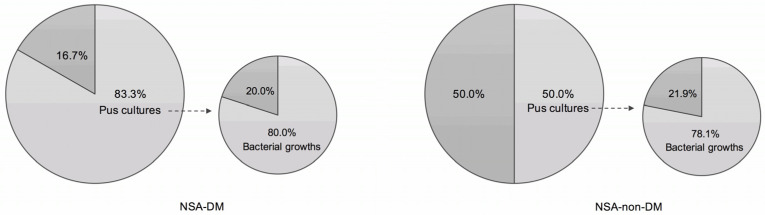
Bacterial culture rate and culture-positive rate.

The bacterial cultures were performed in 83.3% and 50% of patients in the NSA-DM and NSA-non-DM groups, respectively. The positive culture rates were 80% and 78.1% in DM and non-DM patients, respectively. The cultured bacteria were classified into facultative anaerobes or aerobes and anaerobes (Figure 2).

### 3.4. Number of Bacteria Isolates in NSA

**Figure 3:**
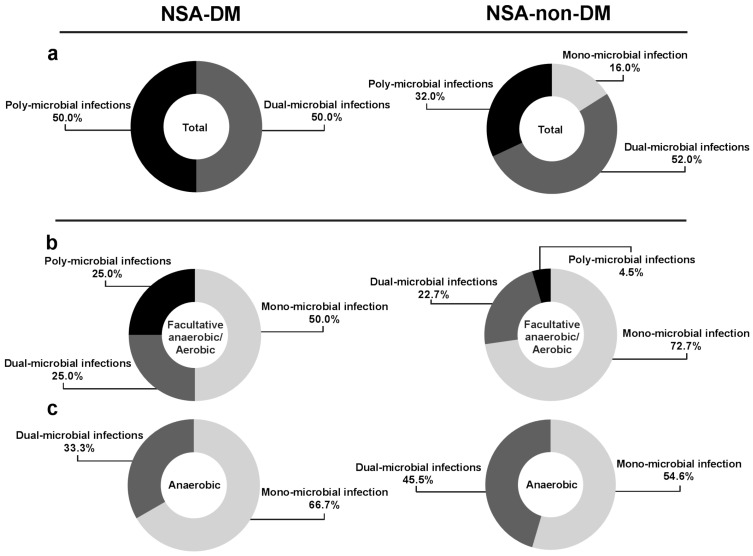
Number of bacteria isolates responsible for NSA infection (**a**) the distribution of the bacterial number for NSA; (**b**) the distribution of the bacterial number for NSA in facultative anaerobes; (**c**) The distribution of the bacterial number for NSA in aerobes and anaerobes.

We analyzed the compositions of bacterial infections of each patient with DM-NSA and non-DM-NSA and classified the results into three groups: mono-microbial, dual-microbial and poly-microbial infections. In NSA-DM, poly-microbial and dual-microbial infections accounted for half of all cultured bacteria. The NSA-non-DM group showed poly-microbial infections (32%), dual-microbial infections (52%) and mono-microbial infections (16%) (Figure 3a). All isolated bacteria were sub-grouped as facultative anaerobes or aerobes and anaerobes. The distribution of the bacterial number for NSA in each subgroup was presented in Figure 3b,c. In both groups, facultative anaerobic or aerobic infection was dominated by mono-microbial infection (mono-microbial infection of NSA in DM: 50%; in non-DM: 72.7%). Similarly, mono-microbial infection was the majority of anaerobic infections in both groups (mono-microbial infection of NSA in DM: 66.7%; in non-DM: 54.6%).

### 3.5. Top Three Genera for NSA

**Figure 4:**
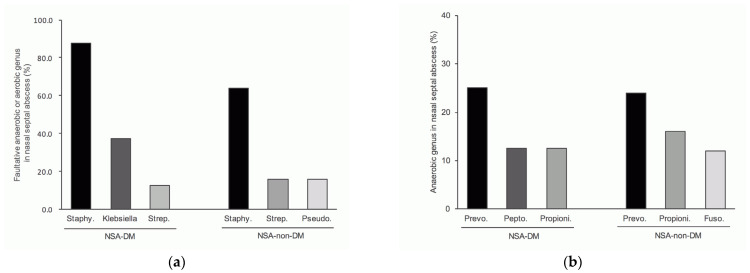
Top three most frequent genera in NSA-DM and NSA-non-DM: (**a**) top three most frequent genera of facultative anaerobes and aerobes (**b**) top three most frequent genera of anaerobes. Abbreviations: Staphy., *Staphylococcus*; Strep., *Streptococcus*; Pseudo., *Pseudomonas*; Prevo., *Prevotella*; Pepto., *Peptostreptococcus*; Propioni., *Propionibacterium*; Fuso., *Fusobacterium*.

The top three common genera of facultative anaerobic or aerobic bacteria in the group of NSA-DM were *Staphylococcus* (87.5%), *Klebsiella* (37.5%) and *Streptococcus* (12.5%), and NSA-non-DM were *Staphylococcus* (64%) *Streptococcus* (16%) and *Pseudomonas* (16%) (Figure 4a).

In the NSA-DM group, the top three most frequent anaerobes are *Prevotella* (25%), *Peptostreptococcus* (12.5%) and *Propionibacterium* (12.5%). Among the group of NSA-non-DM were *Prevotella* (24%) *Propionibacterium* (16%) and *Fusobacterium* (12%) (Figure 4b).

### 3.6. Top Three Species for NSA

Some bacterial cultures of NSA were Coagulase-negative staphylococcus (CoNS). However, generally, we do not treat CoNS as the primary pathogen in clinical practice [15,16]. Therefore, we excluded the CoNS for analysis.

**Figure 5:**
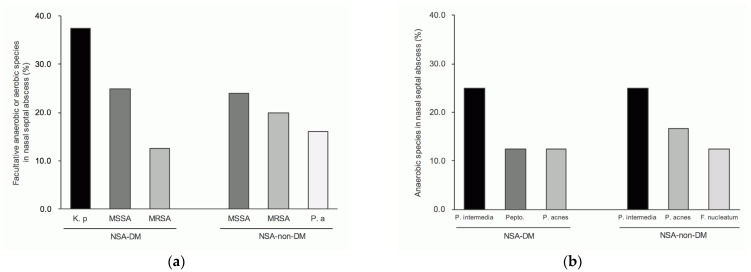
Top three most frequent species in NSA-DM and NSA-non-DM: (**a**) top three most frequent species of facultative anaerobes and aerobes; (**b**) top three most frequent species of anaerobes. Abbreviations: K. p, *Klebsiella pneumoniae*; MSSA, methicillin-sensitive *Staphylococcus aureus*; MRSA, methicillin-resistant *Staphylococcus aureus*; P. a, *Pseudomonas aeruginosa*; P. intermedia, *Prevotella intermedia*; Pepto., *Peptostreptococcus* species; P. acnes, *Propionibacterium acnes*; F. nucleatum, *Fusobacterium nucleatum*.

Among the patients with NSA-DM, the top three most common bacteria were *Klebsiella pneumonia* (37.5%), Methicillin-sensitive *Staphylococcus aureus* (MSSA; 25%) and Methicillin-resistant *Staphylococcus aureus* (MRSA; 12.5%). In the NSA-non-DM group, the top three were MSSA (24%), MRSA (20%) and *P. aeruginosa* (16%) (Figure 5a).

In NSA-DM patients, the most frequently occurring anaerobic bacteria were *Prevotella intermedia* (25%), *Peptostreptococcus* species (12.5%) and *Propionibacterium acnes* (12.5%). In NSA-non-DM patients, the top three were *P. intermedia* (25%), *P. acnes* (16.7%) and *Fusobacterium nucleatum* (12.5%) (Figure 5b).

### 3.7. Prognosis

Among these NSA-DM patients, 10/12 (83.3%) were hospitalized, which was higher than NSA-non-DM patients (42/64, 65.6%). Among the hospitalized patients, DM patients took a more extended hospital stay than non-DM patients (days in the hospital, DM vs. non-DM: 32.8 ± 28.7 vs. 12.3 ± 17.3, *p* = 0.054). Moreover, both groups had no meningitis or encephalitis and no death during the NSA treatment. None of the patients operated to repair a perforated nasal septum.

## 4. Discussion

Despite being a rare disease, NSA can lead to catastrophic complications, including intracranial infection, nasal septal perforation and a saddle nose [4,17]. Therefore, more specific and appropriate antibiotics must be administered to shorten the time to cure and reduce complications. Furthermore, sometimes adequate pus samples are not available for bacterial culture in clinical practice, and bacterial culture may not always yield results (Figure 2). Therefore, a complete understanding of the pathogenic bacterial composition of NSA is essential for clinicians to select antibiotics.

Although most NSAs are caused after trauma or nasal surgery [2,5], some evidence indicated DM could predispose to spontaneous NSA [5,6]. Our previous study demonstrated that diabetic patients have 1.4-fold higher risk of NSA than the non-DM group and a 2.1-fold higher risk in DM patients with a history of nasal septal surgery [1]. Therefore, in our analysis of CGRD, adequate NSA cases were included to explore the bacterial composition of NSA in DM and non-DM patients. The research results have provided an essential reference for clinicians in selecting antibiotics.

Clinicians mainly targeted the facultative anaerobes or aerobes. Among the culture-positive samples, the majority of facultative anaerobes or aerobes were mono-microbial infections (DM vs. non-DM: 50% vs. 72.7%) (Figure 3). Similarly, mono-infection was also predominant anaerobes in both groups (DM vs. non-DM: 66.7% vs. 54.6%). Therefore, antibiotics for NSA should be selected based on the common pathogenic facultative anaerobes or aerobes and anaerobes to improve the clinical therapeutic effect. Moreover, compared with NSA-non-DM patients, NSA-DM patients had a significantly higher proportion of poly-microbial infection in facultative anaerobes or aerobes (DM vs. non-DM: 25% vs. 4.5%). Therefore, the physician should consider the possibility of mixing multiple bacterial and drug-resistant bacterial infections in NSA-DM patients who do not respond to the treatment.

Previous studies have shown that the main pathogenic species of NSA is *S. aureus* [18,19,20]. Similarly, in our NSA-non-DM group, the top two species with the highest frequency were MSSA and MRSA, indicating the high reliability of our database-based research. Among the patients with DM and NSA, the leading pathogen was *K. pneumoniae*, followed by MSSA and MRSA. Moreover, the main anaerobic bacteria were *P. intermedia*. Therefore, in these patients, the empirical antibiotic should cover *K. pneumoniae* and *P. intermedia*, but MSSA and even MRSA infection may need to be considered in treatment failure cases. In the group of non-DM-NSA, the main pathogen is MSSA, followed by MRSA and *P. aeruginosa*, and the anaerobic bacteria are also dominated by *P. intermedia*. Therefore, for NSA-non-DM patients, antibiotics can be considered for MSSA and *P. intermedia* at first. Still, if the treatment response is inadequate, MRSA and *P. aeruginosa* infections need to be considered for the timely replacement of antibiotics.

Previous studies investigated the relationship between DM and *K. pneumoniae* infection in deep neck infection and liver abscesses [7,21,22]. The hyperglycemic status might weaken the intracellular killing ability of peripheral blood mononuclear cells by reducing cytokine and chemokine production and impairing neutrophilic functions and complement activation [22,23]. Previous studies have also observed a higher trend of gram-negative bacilli colonization, such as *K. pneumoniae*, at the upper respiratory tract in DM patients than in non-DM patients [24]. Diabetic patients with poor blood sugar control and high glycated hemoglobin levels are more susceptible to getting *K. pneumoniae* infection than those without hyperglycemic disorders [25]. However, the relationship between DM and *K. pneumoniae* still needs more research to investigate.

Compared with non-DM patients, DM patients had a higher proportion of needing hospitalization for NSA treatment. They had a longer duration of hospitalization, leading to more difficulties in treating the NSA-DM patients. Moreover, none of the patients in the two groups received nasal septal repair surgery after NSA treatment. However, we only reviewed the medical records of the CGRD database, and these patients may have undergone nasal septal repair in other hospitals.

**Figure 6:**
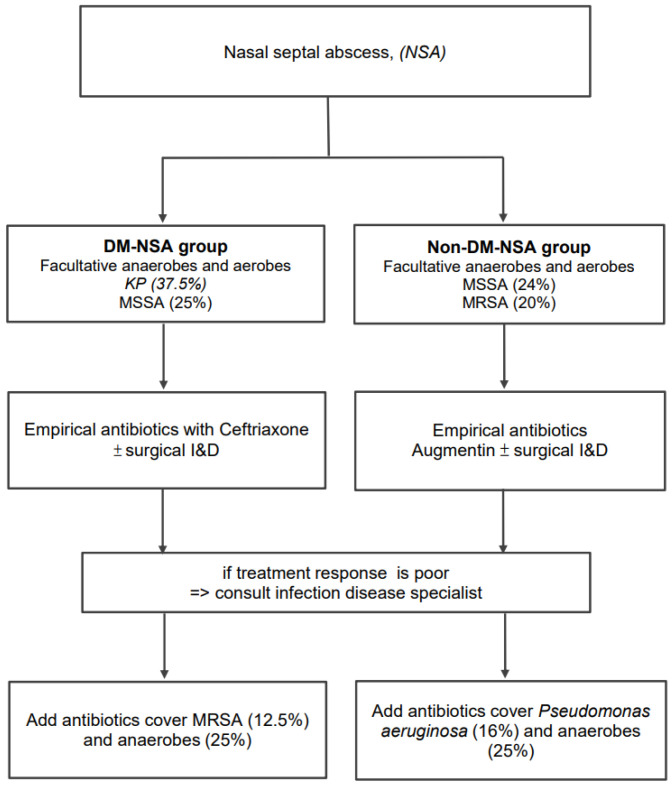
The flowchart for managing a nasal septal abscess. Abbreviations: DM, diabetes mellitus; I&D, incision and drainage.

We propose an algorithm for treating NSA based on current evidence (Figure 6). Because the main bacteria are methicillin-sensitive Staphylococcus aureus (MSSA; 24%) and MRSA (20%), we recommend augmentin (amoxicillin–clavulanic acid) for treating NSA patients without DM. While we recommend third-generation cephalosporins such as ceftriaxone as initial empirical antibiotics in NSA patients with DM, the main pathogens are Klebsiella pneumonia (37.5%) and MSSA (25%). If the response to treatment is poor and the culture results are still unavailable, the treatment can be modified to include Pseudomonas aeruginosa (16%) and anaerobes (25%) in patients without DM and MRSA (12.5%) and anaerobes in patients with DM. A specialist in infectious diseases should also be consulted.

This study has several strengths. We gathered cases from all hospitals in Chang Gung medical system to analyze the bacterial composition of NSA. Compared with previous studies [2,6], our analysis included a more significant number of cases. Additionally, our study is the first to analyze the bacterial composition of DM patients with NSA, which can provide an essential reference for clinicians for choosing antibiotics in patients with NSA and DM. Moreover, the hospitals in the Chang Gung medical system are widely distributed in major administrative regions of Taiwan, and 14% of the total population in Taiwan accepts medical treatment in the Chang Gung medical system [8]. Some validation studies have reported that using the CGRD for research can represent [26,27].

Our study has several limitations. Regardless of the DM, bacterial culture results may be diverse (Figure 2); for example, two facultative anaerobes or aerobes may be co-infected in an NSA case. In the study, we integrated the bacterial culture results of each patient, then calculated the frequency of each genus or species and compared the top three for analysis. However, the actual condition of bacterial infections in clinical practice may be more complex. The results of this study could only represent the frequency of these pathogenic bacteria in NSA to provide a reference for physicians for antibiotics selection. Besides, the percentage of bacterial cultures in NSA ranged from 50% to 83.3%, and the culture-positive rates ranged from 78.1% to 80.0% (Figure 2); therefore, the bacterial analysis could not represent a complete bacterial spectrum of NSA. Finally, we were unable to evaluate the impact of nasal allergy, chronic rhinosinusitis, intranasal steroid use, and smoking history in this study. Future research should look into whether these are independent risk factors.

## 5. Conclusions

When treating NSA in diabetic patients, clinicians should choose empirical antibiotics for *K. pneumoniae* and *P. intermedia* and consider the possibility of MSSA and MRSA infection in treatment failure cases. When treating patients with NSA-non-DM, MSSA and *P. intermedia* should be considered first, bearing in mind the possibility of MRSA and *P. aeruginosa* infection.

## Figures and Tables

**Table 1:** Demographic characteristics and medical comorbidities.

Variables	NSA-DM		NSA-Non-DM		*p*-Value ^†^
	N = 12		N = 64		
	n	%	n	%	
Gender					0.110
Male	10	83.3	36	56.3	
Female	2	16.7	28	43.8	
Age (years)					0.060
<50	4	33.3	41	64.1	
≥50	8	66.7	23	35.9	
Comorbidities					
HTN	6	50.0	4	6.3	0.001
CVA	4	33.3	1	1.6	0.002
CAD	1	8.3	3	4.7	0.505
COPD	1	8.3	2	3.1	0.407

^†^ Pearson’s chi-squared tests and Fisher exact tests. Abbreviations: NSA, nasal septal abscess; DM, diabetes mellitus; HTN, hypertension; CVA, cerebrovascular accident; CAD, coronary arterial disease; COPD, chronic obstructive pulmonary disease.

## Data Availability

The data presented in this study are available on request from the corresponding author.
